# The Permutation Distancing Test for dependent single-case observational AB-phase design data: A Monte Carlo simulation study

**DOI:** 10.3758/s13428-023-02167-5

**Published:** 2023-08-01

**Authors:** Anouk Vroegindeweij, Linde N. Nijhof, Patrick Onghena, Elise M. van de Putte, Sanne L. Nijhof, Jan Houtveen

**Affiliations:** 1 Department of Pediatric Rheumatology/Immunology, Wilhelmina Children’s Hospital, University Medical Center Utrecht, Utrecht University, Utrecht, Netherlands; 2 Department of Pediatrics, Wilhelmina Children’s Hospital, University Medical Center Utrecht, Utrecht University, Utrecht, The Netherlands; 3 Faculty of Psychology and Educational Sciences, KU Leuven, Leuven, Belgium; 4 Altrecht Psychosomatic Medicine, Zeist, The Netherlands

**Keywords:** Autocorrelation, Monte Carlo simulation, Permutation, Permutation distancing test, Single-case observational design

## Abstract

The Permutation Distancing Test (PDT) is a nonparametric test for evaluating treatment effects in dependent single-case observational design (SCOD) AB-phase data without linear trends. Monte Carlo methods were used to estimate the PDT power and type I error rate, and to compare them to those of the Single-Case Randomization Test (SCRT) assuming a randomly determined intervention point and the traditional permutation test assuming full exchangeability. Data were simulated without linear trends for five treatment effect levels (– 2, – 1, 0, 1, 2), five autocorrelation levels (0, .15, .30, .45, .60), and four observation number levels (30, 60, 90, 120). The power was calculated multiple times for all combinations of factor levels each generating 1000 replications. With 30 observations, the PDT showed sufficient power (≥ 80%) to detect medium treatment effects up to autocorrelation ≤ .45. Using 60 observations, the PDT showed sufficient power to detect medium treatment effects regardless of autocorrelation. With ≥ 90 observations, the PDT could also detect small treatment effects up to autocorrelation ≤ .30. With 30 observations, the type I error rate was 5–7%. With 60 observations and more, the type I error rate was ≤ 5% with autocorrelation < .60. The PDT outperformed the SCRT regarding power, particularly with a small number of observations. The PDT outperformed the traditional permutation test regarding type I error rate control, especially when autocorrelation increased. In conclusion, the PDT is a useful and promising nonparametric test to evaluate treatment effects in dependent SCOD AB-phase data without linear trends.

**Supplementary Information:**

The online version contains supplementary material available at 10.3758/s13428-023-02167-5.

## Introducing the Permutation Distancing Test for dependent single-case observational AB-phase design data: A Monte Carlo simulation study

To study treatment effect in one or more individuals, single-case study designs can be implemented (Barlow et al., 2008; Morley, 2017). In contrast to between-group intervention studies which usually only include a pre- and post-intervention measurement, single-case studies incorporate ongoing measurement (Kazdin, 2021). The most basic form of a single-case design is the AB-phase design, in which continuously repeated measurement is implemented throughout a baseline period (Phase A) and the following intervention period (Phase B) (Michiels & Onghena, 2019; Morley, 2017; Tate & Perdices, 2019).

In AB-phase designs, Phase B can start in two different ways. The way Phase B starts, makes a difference in how the time series data should be analyzed afterwards. The first way is by implementing a randomized starting point of Phase B. This design choice fits with Single-Case Experimental Designs (SCED) and has the advantage that it compensates for the lack of intervention reversal phases. Assigning an individual to a randomized starting point increases the AB-phase design’s internal validity (Edgington, 1996; Kratochwill & Levin, 2014; Michiels & Onghena, 2019) and makes it possible to analyze the dependent repeated measurement data with the Single-Case Randomization Test (SCRT) (Bulté & Onghena, 2008; Edgington, 1975, 1980). The SCRT compares the observed test statistic (i.e., mean or median difference between Phases A and B) with the test statistics distribution that would follow from all other possible starting points of Phase B, ultimately determining the statistical significance of the observed test statistic. The second way to start Phase B, is to observe it as a natural event. The starting point can either be fixed or varied across individuals, but cannot be experimentally assigned. This design choice fits with Single-Case Observational Designs (SCOD) and might be preferred when assigning a randomized starting point is considered unethical or impossible (Nikles et al., 2021). The disadvantage of this approach is the lower internal validity, restricting interpretation of the treatment effect whenever a change over time is observed.

A nonparametric test for SCOD AB-phase data with dependent observations, to determine the statistical significance of any difference between Phase A and Phase B, is still lacking. To accomplish this, we propose a randomization test for SCODs that is analogous to the randomization test for SCEDs and that takes into account the serial dependency between the repeated measurements in a single-case AB-phase design. We developed this test for AB-phase data without linear trends as an adaptation of the traditional permutation test, and we call it the Permutation Distancing Test (PDT).

In the following sections, we will first explain the concept of the PDT and demonstrate the test procedure with real dependent SCOD AB-phase data without linear trends. Next, we will investigate the type I error rate and the statistical power of the PDT and compare it to the type I error rate and statistical power of the SCRT and traditional permutation test with a Monte Carlo simulation study.

### Concept of the Permutation Distancing Test

The PDT for dependent SCOD AB-phase data without linear trends is an adaptation of the traditional permutation test (Berry et al., 2021; Box & Andersen, 1955; Good, 2005; Odén & Wedel, 1975). Both tests are nonparametric. Yet, the traditional permutation test examines the null hypothesis of two independent groups that have identical distributions of observations (Odén & Wedel, 1975; Onghena, 2018), whereas the PDT tests the null hypothesis of a single individual having identical distributions of observations over Phases A and B whilst dealing with dependency of observations.

In the traditional permutation test, the null hypothesis is tested by randomly reshuffling the observations in new orders, ultimately reproducing all possible rearrangements of the observed data. These rearrangements are called permutations. For each permutation, the test statistic is determined, such as the mean difference between groups (*Ῡ1 - Ῡ2*) or the median difference between groups ($\bar{M}$*1 –* $\bar{M}$*2*). Next, the probability of obtaining the observed test statistic or a more extreme value is computed by dividing the number of equal or more extreme values by the total number of possible permutations (Berry et al., 2011; Nichols & Holmes, 2002; Winkler et al., 2014). The PDT follows a similar procedure with the mean or median difference between two phases as test statistic (*ῩA - ῩB* or $\bar{M}$*A –* $\bar{M}$*B*) whilst taking care of dependency by applying stepwise down-sampling while using all available observations (as further explained below).

The PDT is available as an open-source *R*-package (see https://CRAN.R-project.org/package=pdt). To perform the PDT, three input variables are required. The first variable is *x*, a factor indicating whether the observation belongs to Phase A or to Phase B. Depending on the kind of treatment provided, it can be hypothesized that an individual improves directly at the start of Phase B, or with a delay (a lag). In case of the latter, Phase B should indicate the moment of hypothesized improvement after the start of treatment. The second variable is a numeric time marker, which can be referred to as *x values* (i.e., the time value to variable *x*). The last variable is *y*, also numeric, representing the observed value of the outcome variable. The PDT needs an equidistant dataset, meaning that all time markers with corresponding *y* observations should be present and in the right order. If this is not the case, the data will be made equidistant by (1) including missing time markers and setting the corresponding *y* values to missing values (*NAs*) and (2) shifting or mean-aggregating the *y* values of duplicate time markers. Then, the data can be plotted for visual inspection and preliminary estimation of the treatment effect. If the data are considered free of linear trends, the permutations can be performed.

The PDT takes care of dependency between observations. With dependency, we refer to autocorrelation (*ar*) (Bolger & Laurenceau, 2013). In other words, the extent to which an individual's measurement response in the future is connected to its prior measurement response (Bolger & Laurenceau, 2013; Du & Wang, 2018). Measurements closer in time are presumably more similar to one another than measurements further apart (Bolger & Laurenceau, 2013). As the traditional permutation test has been designed to analyze cross-sectional data, it cannot take *ar* into account. Ignoring *ar* in (intensive) longitudinal data often leads to effect estimations with standard errors that are too narrow and test statistics that are too large, increasing the likelihood of type I errors (Bolger & Laurenceau, 2013). The PDT takes care of *ar* through stepwise down-sampling based on the assumption that a larger temporal distance (more time) between observations reduces serial dependency. This is done by reproducing all possible permutations in subsets of the observed data with an induced temporal distance, while varying the degree of temporal distance between the observations (*k*) ascendingly. The down-sampling (or distancing) is realized by replacing (*k-1*) intermediate *y* values with *NAs*. The value of *k* increases from 1 up to the maximum of *k* (*k_max*)*.* In other words, the PDT creates subsets of the data with identical *k*, *k*=1, 2, 3, … *k_max* distance, in which *k-1* is the number of *NAs* between observations and *k_max* is determined by the number of observations the dataset has. Note that the permutations will be performed on subsets with zero *NAs* between the observations if *k* = 1 (i.e., the original equidistant dataset). To decide which temporal distance *k* should be interpreted as favored, a Ljung-Box test (Burns, 2002) is used to indicate at which distance of *k* the serial *ar* is no longer significantly present in the data (at a significance level of 5%). The PDT runs until that point of *k* is reached and returns for each tested *k* the Ljung-Box test results and permutation test results. For the favored *k*, the PDT separately returns the observed test statistic (*ῩA - ῩB)* or ($\bar{M}$*A -* $\bar{M}$*B*).

The PDT also returns effect sizes which are based on Cohen’s *d* effect size $\frac{ \mid A-B \mid }{pooled\ SD}$ , with *| A – B |* being the mean or median difference^1^ between Phases A and B. The effect size indicates the difference in standard deviation units between both phases. Note that the effect size of the favored *k* should approach the ‘original’ effect size (based on the original equidistant dataset in which *k* = 1). Following the proposed classification for single-case effect sizes similar to Cohen’s *d*, effect sizes with a value of 0.00–0.99 are interpreted as small, 1.00–2.49 as medium and ≥ 2.50 as large (Harrington & Velicer, 2015). Together with the other PDT output, the effect size of the favored k can be used to clinically explore individual differences in treatment effects across individuals, or to compare different treatments over time in a single individual.

### Demonstration of the Permutation Distancing Test: FITNET-plus study

To demonstrate the PDT procedure, we use a real single-case SCED AB-phase dataset with relatively few observations and show how it would be analyzed as SCOD data. The data come from an adaptation of the FITNET (Fatigue In Teenagers on the interNET) trial (S. L. Nijhof et al., 2012), named FITNET-plus. In the FITNET-plus study, nine chronically fatigued adolescents with a chronic medical condition were observed before, during, and after the start of internet-delivered Cognitive Behavioral Therapy (I-CBT) (L. N. Nijhof et al., 2023). During all weeks, adolescents completed the Checklist Individual Strength-8 questionnaire (CIS-8), which measures fatigue severity on a scale of 8 to 56, with higher scores indicating more fatigue severity (L. N. Nijhof et al., 2023; Worm-Smeitink et al., 2017). The CIS-8 total score was the primary outcome variable used to evaluate treatment effect, with a validated cut-off score of > 39 to indicate severe fatigue (L. N. Nijhof et al., 2023; Worm-Smeitink et al., 2017). The presented data in the following sections all belong to one participant of the FITNET-plus intervention (see Table 1). Performing the SCRT for SCED data yielded no significant treatment effect (*p* = 0.194). However, because the SCRT has on average ≤ 50% power in case of 30 measurement observations (Michiels & Onghena, 2019), it is possible that the SCRT has missed a treatment effect that was actually present. We will therefore re-test the same dataset with the PDT.

**Table 1:** The input data of the FITNET-plus study example

***time***	Week -11	Week -10	Week -9	Week -8	Week -7	Week -6	Week -5	Week -4	Week -3	Week -2
***x***	A	A	A	A	A	A	A	A	A	A
***y***	48	43	35	43	42	47	44	46	47	47
***time***	Week -1	Week 0	Week 1	Week 2	Week 3	Week 4	Week 5	Week 6	Week 7	Week 8
***x***	A	B	B	B	B	B	B	B	B	B
***y***	50	48	47	41	30	35	38	32	28	26
***time***	Week 9	Week 10	Week 11	Week 12	Week 13	Week 14	Week 15	Week 16	Week 17	Week 18
***x***	B	B	B	B	B	B	B	B	B	B
***y***	35	38	40	30	34	37	38	42	40	46
***time***	Week 19	Week 20	Week 21	Week 23	Week 24	Week 25	Week 26			
***x***	B	B	B	B	B	B	B			
***y***	42	34	27	29	28	21	24			

## Input variables

In our FITNET-plus example, the input data look as follows:

The time markers show that the first observation took place 11 weeks before the start of treatment. The last observation took place 26 weeks after the start of treatment. Variable *x* confirms this by indicating whether the data point belongs to Phase A, which is the baseline before start of treatment, or to Phase B, which is the period after start of treatment. In case of a predicted delay in the improvement, the transition from Phase A to Phase B can be delayed. During each week, the primary outcome variable *y* was measured, which was the CIS-8 total score. In the first 2 weeks of the study, the participant had a CIS-8 total score of 48 and 43, respectively. Those scores indicated that he or she suffered from severe fatigue, which continued during the following weeks. After the start of treatment, a decline in fatigue is hypothesized.

## Equidistance

Equidistance is needed for the process of distancing. In the FITNET-plus example presented in Table 1, the time markers *week -11* are followed up by *week -10*, *week -9*, *week -8* and so on, but *week 22* is missing. This makes the dataset not equidistant. The PDT can solve this by including *week 22* in the sequence of time markers and adding *NA* to the corresponding *y* observation. Then, the returned data will look as presented in Table 2.

**Table 2:** Missing time markers in the FITNET-plus study example

***time***	…	Week 18	Week 19	Week 20	Week 21	Missing Week 22	Week 23	Week 24	Week 25	Week 26	
***x***	…	B	B	B	B	B	B	B	B	B	
***y***	…	46	42	34	27	*NA*	29	28	21	24	

Datasets may also include duplicate time markers which also makes it not equidistant. This can happen with experience sampling methodology (ESM) due to a technical error and/or because the observations followed each other closely (e.g., when completion of the previous questionnaire was delayed). The presented FITNET-plus participant does not have duplicate time markers, but for illustrative purposes, we have added *week 23* as duplicate to Table 3.

**Table 3:** Duplicate time markers in the FITNET-plus study example

***time***	…	Week 18	Week 19	Week 20	Week 21	Missing 22	Week 23	Week 23	Week 24	Week 25	Week 26
***x***	…	B	B	B	B	B	B	B	B	B	B
***y***	…	46	42	34	27	*NA*	29	27	28	21	24

The PDT can remove the first duplicate *week 23* and shift its *y* value to *week 22* if that value is *NA*, or remove the last duplicate *week 23* and shift its *y* value to *week 24* if that value is *NA,* to compensate for a respectively early or delayed observation. If time markers are duplicates and cannot be shifted, then the PDT replaces the *y* value of this time marker with the mean-aggregated CIS-8 total scores of the corresponding duplicates.

## Visual inspection of the data

**Fig. 1:**
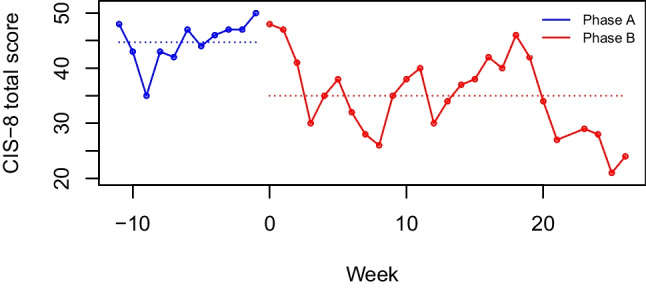
Permutation Distancing Test plot of the FITNET-plus example. *Note.* Outcome of the FITNET-plus intervention was 'fatigue severity' as measured by the CIS-8 questionnaire. Higher total scores indicate more fatigue severity. The CIS-8 also has a validated cut-off score of > 39 to indicate severe fatigue. Phase A is presented in blue. Phase B is presented in red. The horizontal lines represent the mean of each phase. In case of skewed distributions in the dataset, median lines can be computed and visualized in the plot instead

Figure 1 (created with the PDT plot option for visual inspection) shows the plotted data of the FITNET-plus study example with the outcome observations throughout Phases A and B. The figure shows no linear trends, indicating that we can use the PDT on this dataset. Inspection of the mean difference between Phases A and B (i.e., the horizontal lines), suggests that the difference is large enough to find a significant treatment effect. With the PDT, we will test whether the visually observed difference between phase A and B is indeed statistically significant.

## Permutation with equidistance

After visual inspection of the data, the PDT can be performed. With the observations presented in Table 1, all possible subsets (*l = 1 to k*) of data will be created for each ascending distance *k* between the observations. In the FITNET-plus example, we used the default option for *k_max* and the PDT ran until *k* = 3, indicating there was no longer significant *ar* present at a distance of two *NAs* between the observations. Thus, in the FITNET-plus example, permutation tests would be performed on the subsets as presented in Table 4.

**Table 4:** The three possible subsets (l) with k = 3 in the FITNET-plus study example

*l=1:*	**Y**	48	*NA*	*NA*	43	*NA*	*NA*	44	*NA*	*NA*	47	…
*l=2:*	**Y**	*NA*	43	*NA*	*NA*	52	*NA*	*NA*	46	*NA*	*NA*	…
*l=3:*	**Y**	*NA*	*NA*	35	*NA*	*NA*	47	*NA*	*NA*	47	*NA*	…

## Test results

Table 5 summarizes the output of the permutation distancing test for the FITNET-plus example. At *k* = 3, the observed test statistic (*ῩA - ῩB*) had a value of 9.727 with a *p* value of 0.026. The effect size at *k* = 3 was 1.691, which we interpret as a medium effect size (Harrington & Velicer, 2015). It was somewhat smaller than the original effect size of 1.725 based on the *k* = 1 subsets (i.e., from the original equidistant dataset). Altogether, the results indicate that the FITNET-plus study participant (with alpha = .05) was significantly less fatigued after the start of I-CBT.

**Table 5:** Permutation Distancing Test output for the FITNET-plus study example

Output	*k* = 1	*k* = 2	*k* = 3
Ljung-Box test results			
Autocorrelation	.724	.466	.244
Test statistic X^2^	20.99	4.93	.910
*p* value	0.000	0.007	0.074
PDT results			
Fitted *p* value^a^	0.000	0.006	0.026
Effect size	1.725	1.797	1.691
Observed test statistic (*ῩA - ῩB*)			9.727

The PDT output consists of the Ljung-Box test results indicating at which temporal distance of *k* the serial autocorrelation is no longer significantly present in the data and the PDT results per *k*. The PDT returns the observed test statistic value for the favored *k*. ^a^ = based on fitting a second order regression line through the course of the *p* value as a function of *k*. The predicted *p* value from this equation returns as the fitted *p* value

## Validation of the Permutation Distancing Test: Monte Carlo simulation

### Simulation method

The PDT is developed to analyze dependent SCOD AB-phase data without linear trends. The purpose of the validation study is to demonstrate for which conditions this test has sufficient statistical power (≥ 80%) to detect treatment effects with acceptable low type I error rates (≤ 5%), and to compare the performance of the PDT with those of the SCRT and traditional permutation test in terms of statistical power and type I error rates. We focus on simulated single-case data without linear trends in Phases A and B. Our expectations are as follows:

I. Statistical power of the PDT is expected to increase as the treatment effect or the number of measurement observations become larger and should not be influenced by the level of autocorrelation.
II. If the null hypothesis of no treatment effect is true, the proportion of rejections of the PDT is expected to be smaller than or equal to the significance level (i.e., chance of type I errors ≤ 5%), independently of the level of autocorrelation.
III. The PDT is expected to outperform the SCRT in terms of statistical power in case of relatively few observations.
IV. The PDT is expected to outperform the traditional permutation test in terms of type I error rate control as the level of autocorrelation increases.^2^

Our expectations are tested by means of Monte Carlo simulations. Following Michiels and Onghena (2019), we built on the Huitema–McKean model regression equation (Huitema & McKean, 2000) to generate the Monte Carlo simulation data in R (version 4.1.0) using RStudio, resulting in the modified and simplified equation:

$$Yt = b0 + b2Dt + \varepsilon t, with:$$

*Y*t being the outcome at time *t* = 1, 2, …, *n*A, *n*A+1, … *Ns*=*n*A+*n*B, with *n*A being the number of observations in Phase A, and *n*B being the number of observations in Phase B,

*b*0 being the regression intercept, *b*0 is set to 0,

*b*2 being the regression coefficient for the mean level treatment effect,

*D*t being the dummy variable [value 0,1] indicating the treatment phase at time *t*,

*ε*t being the error at time *t*, sampled from a standard normal distribution or from a first-order autoregressive (AR1) model.

In the Huitema–McKean model regression equation (Huitema & McKean, 2000) *b*1 represents the slope estimate of Phase A. Parameter *b*3 from this equation has been redefined by Michiels and Onghena (2019) to indicate the value of trend in Phase B independent of the level of trend in Phase A. These parameters were set to 0 in the current study. Following Michiels and Onghena (2019), *ε*t was either sampled from a standard normal distribution (AR1 = 0) or from a first-order autoregressive model with different levels of AR1. Note that residuals from a standard normal distribution are equivalent to the residuals with an autocorrelation of 0. Following Michiels and Onghena (2019), *n*A and *n*B (adding up to the total number of observations *N*s) were programmed to vary across the replications to simulate randomization of the start of the treatment phase B; *n*A varies from *n1_limit* to *Ns*-*n1_limit*, and (corresponding) *n*B varies from *Ns*-*n1_limit* to *n1_limit*. This randomization was needed to test the simulated data sets with the SCRT. The factor levels used in this validation study were:

*ar1*: 0, .15, .30, .45, .60
*Ns*: 30, 60, 90, 120
*b2*: -2, -1, 0, 1, 2
*n1_limit*: 5, 10

Ultimately, factorial crossing of all factor levels generated (5 x 4 x 5 x 2 =) 200 combinations of factor levels. The statistical power of the of the PDT, SCRT, and traditional permutation test for each combination of factor levels was calculated by generating 1000 replications, and the proportion of rejected null hypotheses per combination was calculated at a 5% significance level. The statistical power for each combination of factor levels was determined five times (each based on 1000 replications that were different in simulated error terms). In this way, the power for each level per condition was reflected by sufficient independent lines in the results file to compute reliability intervals.

## Results

**Fig. 2:**
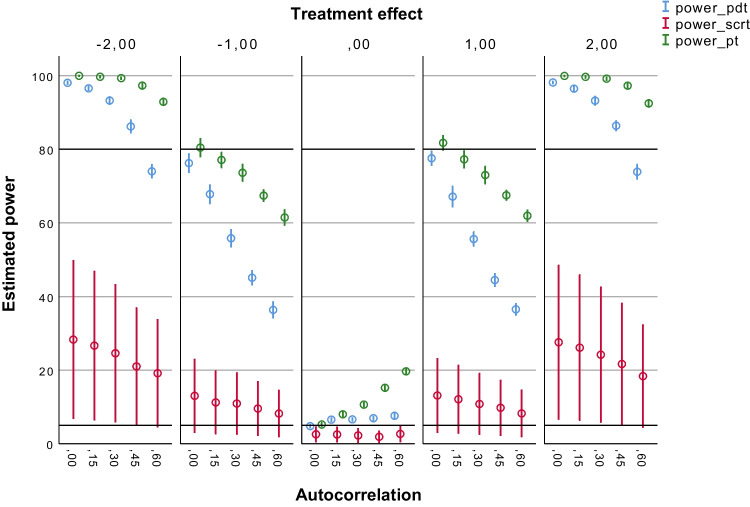
Estimated power of the three tests with 30 observations. *Note.* Average estimated power (with 95% confidence interval) of the Permutation Distancing Test (*blue*), Single-Case Randomization Test (*red*), and traditional permutation test (*green*), without linear trends present in the data and with 30 observations. The *upper black line* indicates 80% power threshold. The *lower black line* indicates 5% power threshold. Treatment effect size is either negative medium (– 2), negative small (– 1), not present (0), positive small (1), or positive medium (2). Level of autocorrelation ranges from null (0) to large (.60)

**Fig. 3:**
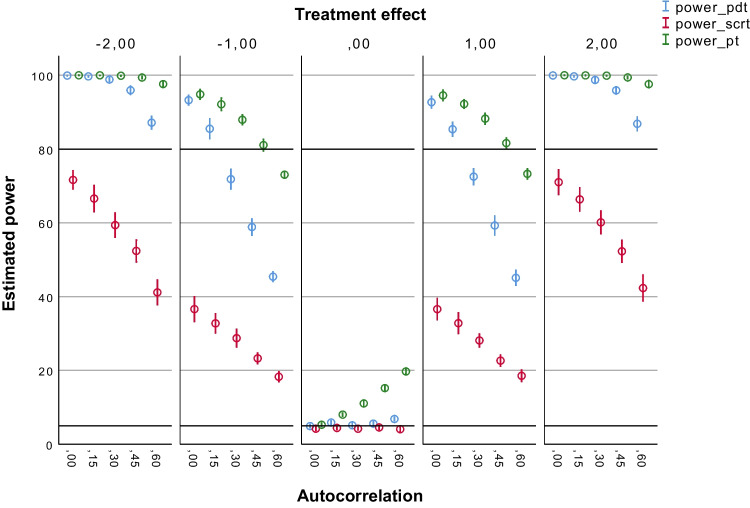
Estimated power of the three tests with 60 observations. *Note.* Average estimated power (with 95% confidence interval) of the Permutation Distancing Test (*blue*), Single-Case Randomization Test (*red*), and traditional permutation test (*green*), without linear trends present in the data and with 60 observations. The *upper black line* indicates 80% power threshold. The *lower black line* indicates 5% power threshold. Treatment effect size is either negative medium (– 2), negative small (– 1), not present (0), positive small (1), or positive medium (2). Level of autocorrelation ranges from null (0) to large (.60)

**Fig. 4:**
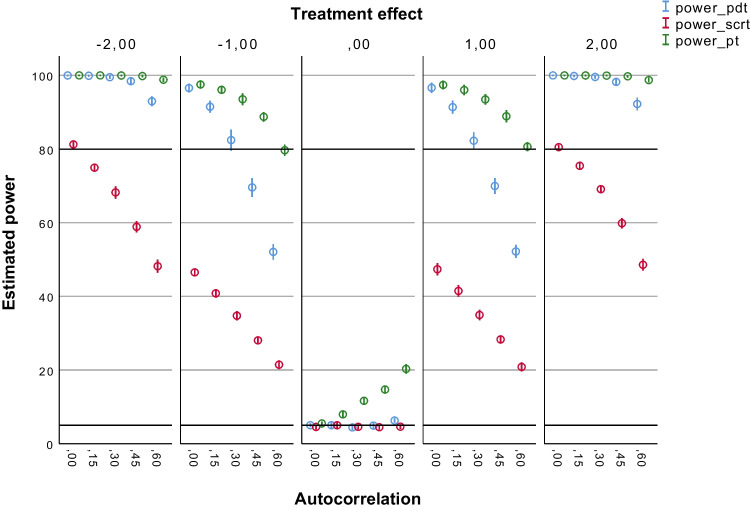
Estimated power of the three tests with 90 observations. *Note.* Average estimated power (with 95% confidence interval) of the Permutation Distancing Test (*blue*), Single-Case Randomization Test (*red*), and traditional permutation test (*green*), without linear trends present in the data and with 90 observations. The *upper black line* indicates 80% power threshold. The *lower black line* indicates 5% power threshold. Treatment effect size is either negative medium (– 2), negative small (– 1), not present (0), positive small (1), or positive medium (2). Level of autocorrelation ranges from null (0) to large (.60)

**Fig. 5:**
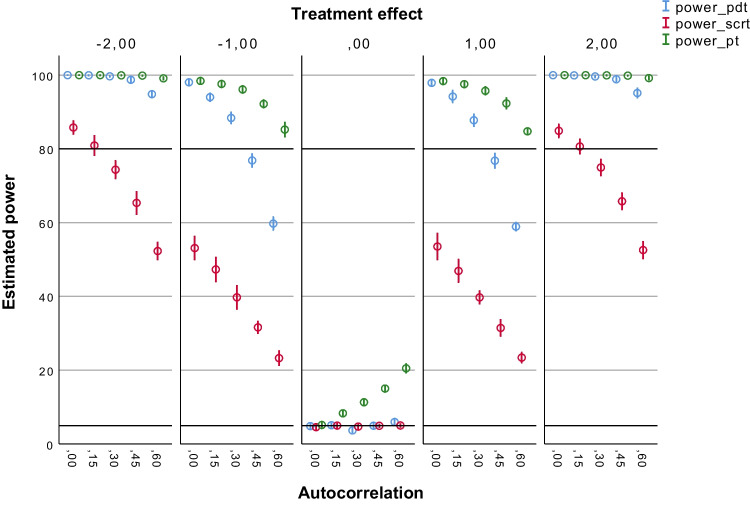
Estimated power of the three tests with 120 observations. *Note.* Average estimated power (with 95% confidence interval) of the Permutation Distancing Test (*blue*), Single-Case Randomization Test (*red*), and traditional permutation test (*green*), without linear trends present in the data and with 120 observations. The *upper black line* indicates 80% power threshold. The *lower black line* indicates 5% power threshold. Treatment effect size is either negative medium (– 2), negative small (– 1), not present (0), positive small (1), or positive medium (2). Level of autocorrelation ranges from null (0) to large (.60)

**Fig. 6:**
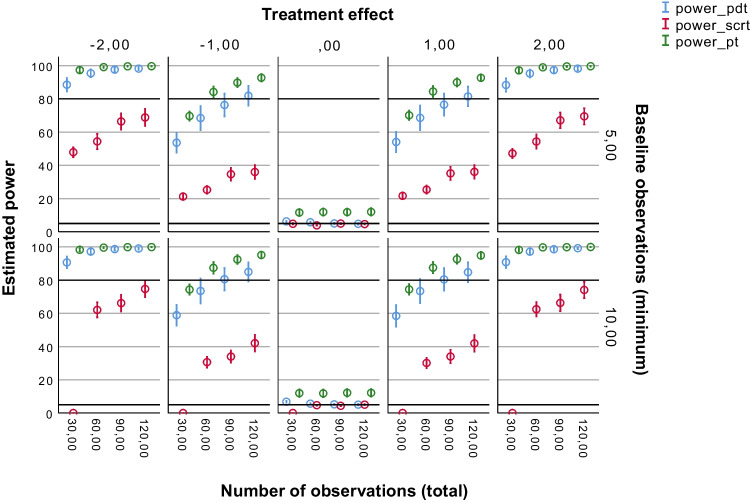
Power of the three tests per minimum number of baseline observations. *Note*. Average estimated power (with 95% confidence interval) of the Permutation Distancing Test (*blue*), Single-Case Randomization Test (*red*), and traditional permutation test (*green*), without linear trends present in the data and with pooled levels of autocorrelation. The *upper black line* indicates 80% power threshold, *lower black line* indicates 5% power threshold. Treatment effect size is either negative medium (– 2), negative small (– 1), not present (0), positive small (1), or positive medium (2). The number of minimum observations during baseline (Phase A) is either 5 or 10. The total number of observations is 30, 60, 90, or 120

Figures 2, 3, 4, 5 and 6 summarize the estimated statistical power results of the three tests under different conditions. As expected, the estimated power results of the PDT were largely determined by the size of the treatment effect (*b*2) and the number of observations (*N*s). Figure 2 shows that the PDT had a power > 80% to detect medium treatment effects (*b*2 = – 2 or 2) up to autocorrelation levels of ≤ .45 with 30 observations. The proportion of type I errors (for *b*2 = 0) was 5% without autocorrelation (*ar1* = 0), and it was slightly above 5%, namely 5–7%, in case autocorrelation was present in the data. With 60 observations (see Fig. 3), the PDT always had a power > 80% to detect medium treatment effects (*b*2 = – 2 or 2) regardless of the level of autocorrelation. Up to autocorrelation levels of ≤ .45, the proportion of type I errors was ≤ 5%. With *ar1* = .60, the proportion was 5–7%. With 90 observations (see Fig. 4), the PDT had a power > 80% to also detect small treatment effects (*b*2 = – 1 or 1) up to autocorrelation levels of ≤ .30. The proportion of type I errors decreased to 5–6% at *ar1* = .60. With 120 observations (see Fig. 5), the statistical power of the PDT improved with 10% in case for small treatment effects with *ar1* levels of ≥ .30. The proportion of type I errors remained 5–6% at *ar1* = .60.

Compared to the PDT, the traditional permutation test had larger power to detect small treatment effects (*b*2 = – 1 or 1), regardless of autocorrelation in the data. However, with the traditional permutation test, the proportion of type I errors (*b*2 = 0) was much larger (i.e., up to 20%) in case of autocorrelation. The SCRT had less power as compared to the PDT for all treatment effects and numbers of observations, but the SCRT always kept the proportion of type I errors below the significance level, even in case of a small number of observations.

The effects of the minimum number of baseline observations (*n1_limit*) on the estimated statistical power results of the PDT, the SCRT, and the traditional permutation test were explored (see Fig. 6). It should be noted, however, that this effect was only of relevance for the SCRT, especially in case of a relatively small number of observations. Effects of *n1_limit* on the power results of the PDT and the traditional permutation test were nonetheless explored as well. As expected, only the SCRT (for *Ns* = 30 and 60) showed differences between the two levels of *n1_limit*.

## Discussion

The PDT has been designed as a nonparametric permutation test to evaluate treatment effects for dependent SCOD AB-phase data without linear trends. The test controls serial autocorrelation through stepwise down-sampling while using all available observations. This is realized by reproducing all possible permutations in subsets of the observed data with an induced temporal distance and varying the degree of temporal distance between the observations ascendingly.

In the present simulation study, we demonstrated the capacity of the PDT for evaluating treatment effects. With 30 observations, sufficient power (≥ 80%) was estimated to detect medium treatment effects up to autocorrelation levels of ≤ .45. With 60 observations, sufficient power was observed to detect medium treatment effects regardless of the level of autocorrelation. With 90 observations and more, sufficient power was estimated to also detect small treatment effects up to autocorrelation levels of ≤ .30. With 30 observations, the type I error rate was only slightly above 5%, namely 5–7%. With 60 observations and more, the type I error rate was generally ≤ 5%.

We compared the statistical power and type I error rate of the PDT with those of the SCRT. The PDT outperformed the SCRT regarding power, especially with a small number of observations (i.e., 30 observations). However, it should be noted that the two tests are developed for different single-case AB-phase designs. The PDT is developed for observational designs (without a randomized intervention starting point), whereas the SCRT is developed for experimental designs (with a randomized intervention starting point). The latter has higher internal validity (Michiels & Onghena, 2019). The SCRT also has tight type I error rate control (Michiels & Onghena, 2019), which we observed in the present simulation study once more. Yet, a relatively large number of observations is required for the SCRT to detect treatment effects with adequate power (Michiels & Onghena, 2019). In the present simulation study, sufficient power for the SCRT was only estimated with 120 observations and low levels of autocorrelation. One may consider using the PDT instead of the SCRT with SCED data in case of too little observations (i.e., less than 120) or larger levels of autocorrelation (i.e., larger than .15). In these scenarios, the PDT can be used to explore potential treatment effects with more statistical power than the SCRT can provide. However, given that the PDT has been developed for SCOD data specifically, it has not been validated to allow causal statements on SCED data. If a cause-and-effect relationship is the main interest of a SCED study, researchers should continue to use the SCRT while increasing the number of observations and the assessment interval.

The performance of the PDT was also compared with that of the traditional permutation test. At first, the traditional test seemed to outperform the PDT in terms of statistical power. However, the traditional permutation test was not designed to handle dependency between observations (Berry et al., 2021; Box & Andersen, 1955; Good, 2005; Odén & Wedel, 1975). That shows when we look at the type I error rate, which ranged between 5 and 22%, depending on the level of autocorrelation, regardless of the number of observations. The type I error rate of the PDT was generally ≤ 5%, and only 5–7% in case of a small number of observations or the presence of the largest autocorrelation level. For the traditional permutation test, it means that the likelihood of a false positive treatment effect was too large. Thus, this test cannot be used reliably for dependent SCOD data. In the presence of autocorrelation, the PDT should be used instead.

As with most other statistical tests, type II errors were more likely to occur with the PDT when a small number of observations was used to detect small treatment effect sizes. To some extent, type II errors can be prevented by increasing the number of observations (Banerjee et al., 2009). This was shown in the present simulation study as well. We also showed that type II errors were partially influenced by the level of dependence between observations. With 30 observations to detect small treatment effect sizes, the type II error rate of the PDT was 22% without any autocorrelation. At the highest level of autocorrelation, the type II error rate was 65%. With 90 observations to detect small treatment effects, the type II error rate already dropped to ≤ 20% up to medium levels of autocorrelation. In practice, one can get the most out of the PDT with 90 observations and more. Then, the type I error rate could reach to 7% with the largest level of autocorrelation but is otherwise ≤ 5%, medium treatment effect sizes can be detected with ≥ 80% power regardless of autocorrelation, and small treatment effect sizes can be detected with ≥ 80% power up to medium levels of autocorrelation.

The PDT has been designed for dependent SCOD AB-phase data without linear trends. If not taken into account, the presence of linear trends can lead to invalidated conclusions regarding single-case treatment effectiveness (Manolov et al., 2019; Parker et al., 2006). This is also the case for the PDT, as findings indicated that the type I and type II error rates became unacceptably large in the presence of trends (see Supplementary Tables 1–4). Linear trends in single-case longitudinal data can be detected through visual inspection or trend estimation techniques (Lobo et al., 2017; Manolov, 2018; Manolov et al., 2023). The PDT *R*-package provides functions to initially inspect trends and to detrend data from Phase A, Phase B, or both phases. Solomon (2014) concluded that procedures to control for trends should be used far more often than currently done. The study by Parker et al. (2006) showed, for instance, that 41% of the 165 investigated datasets required baseline trend control. However, as removing trends can lead to under- or overestimation of the observed treatment effect (Gorsuch, 1983; Manolov, 2018; Manolov et al., 2010; Parker & Brossart, 2003; Shadish et al., 2014; Tarlow, 2017), detrending is not incorporated in the PDT as default option. We recommend not to use the PDT in the presence of trends, and otherwise to interpret the results carefully after detrend functions have been applied. Nonetheless, researchers may consider evaluating results without removing trends based on their knowledge of the context (e.g., when natural recovery during baseline is allowed or if gradual improvement during the treatment phase is expected). In the future, reliable statistical tests for dependent SCOD AB-phase data with linear trends still present in the data may be developed.

The PDT offers single-case *p* values and effect sizes. To generalize these results across participants, one may compute the percentage of subjects with a significant effect, or the pooled effect size across participants. Another generally recommended option is multilevel modelling of fixed-effect estimates across single-case studies (What Works Clearinghouse [WWC], 2020; Ferron et al., 2009), for which nonparametric multilevel tests are available for SCED data (Michiels et al., 2020; Onghena et al., 2018), but, to our knowledge, not for SCOD data yet. Future research may focus on this.

Thus far, we described the use of the PDT in dependent AB-phase SCOD data. In case of more phases (e.g., ABAB-phase or multiple-baseline designs) one may consider splitting the data into separate two-phase comparisons applying Bonferroni correction for multiple testing. A limitation to this approach is that then no omnibus test is performed on the data from the whole design. Furthermore, splitting data into separate specific AB-phase comparisons could introduce bias of data sampling (i.e., inadequate number of observations per condition or inadequate number of effect demonstrations to infer a causal relationship; see Reichow et al., 2018). The SCRT is the preferred test for more complicated designs.

Finally, we validated the PDT with normally distributed continuous data in the current Monte Carlo simulation study. Given the nonparametric nature of the test, the PDT should be robust for non-normal outcomes as well (e.g., measurements on a nominal or ordinal scale including ranks or ratings, but also counts or percentages outcomes; Vrbin, 2022). Additional validation with non-normal distributed dependent data is needed to confirm such applications.

## Conclusions

The present study validated the PDT with Monte Carlo simulation of dependent SCOD AB-phase data without linear trends. This validation demonstrated sufficient statistical power to evaluate medium treatment effects regardless of the level of autocorrelation, and sufficient power for small treatment effects up to medium levels of autocorrelation. The type I error rate was generally ≤ 5% and only slightly above 5% in case of a small number of observations or the highest level of autocorrelation. The PDT can be used for nonparametric testing of a two-phase SCOD dataset with serial dependency, and to explore a potential treatment effect in a SCED-dataset with too little observations for a SCRT-test.

### Supplementary Information

ESM 1 (DOCX 364 kb)

## Footnotes

1 Note with regard to the median difference: it is not common to put a rank-based statistic in the numerator and moment-based statistic in the denominator.

2 Note that the traditional permutation test was not meant to be applied to dependent single-case AB phase data. This test was included in our simulation study to demonstrate what happens when autocorrelation is not taken into account during permutations.
