# Supplementary material for: The Permutation Distancing Test for dependent single-case observational AB-phase design data: A Monte Carlo simulation study
Source: Behav Res Methods. 2023 Aug 1;56(3):2569–80. doi: 10.3758/s13428-023-02167-5 (PMC10991042; doi:10.3758/s13428-023-02167-5)
Supplement: Supplementary file 1 — (DOCX 364 kb) [file 13428_2023_2167_MOESM1_ESM.docx]

**Supplementary Table 1.**

*Estimated power of the three tests with Phase A trend present and 30 observations*


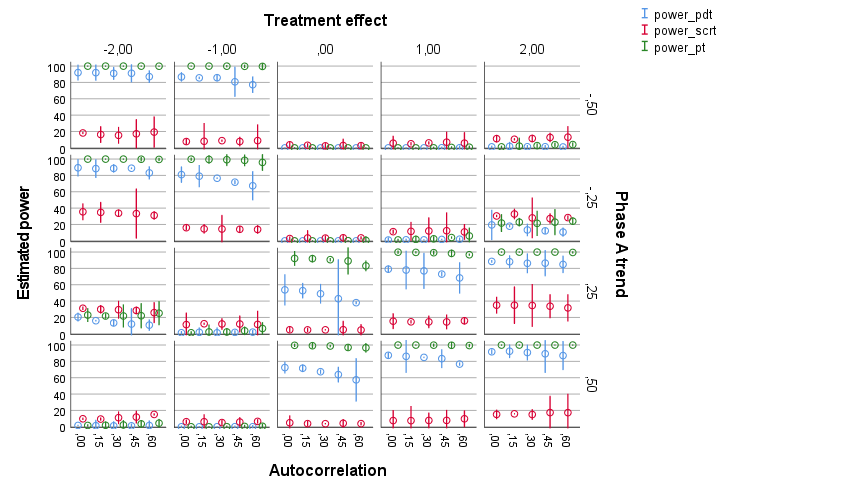


*Note.* Average estimated power (with 95% confidence interval) of the Permutation Distancing Test (blue), Single-Case Randomization Test (red), and traditional permutation test (green), with linear Phase A trends present in the data with 30 observations, per treatment effect size and level of autocorrelation. No Phase B trends were present in the data. To reduce a very long running time the minimum number of baseline observations was set to 5 and each simulation condition was only repeated 2x 1000 times.

**Supplementary Table 2.**

*Estimated power of the three tests with Phase B trend present and 30 observations*


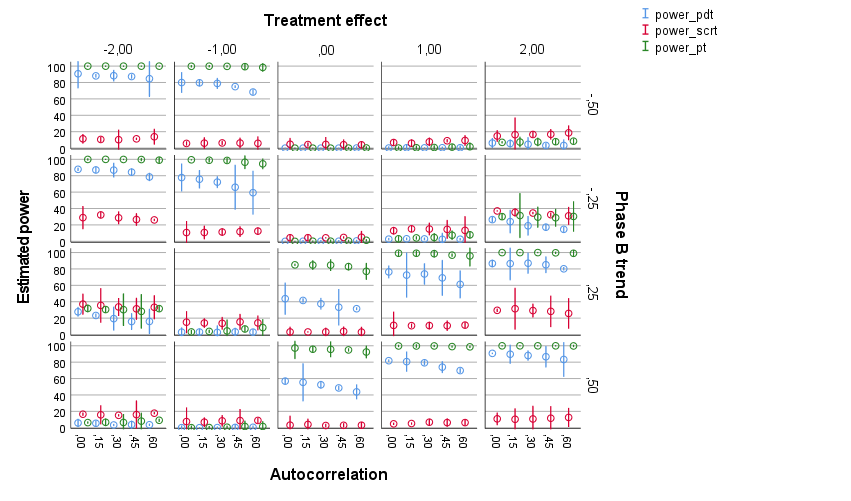


*Note.* Average estimated power (with 95% confidence interval) of the Permutation Distancing Test (blue), Single-Case Randomization Test (red), and traditional permutation test (green), with linear Phase B trends present in the data with 30 observations, per treatment effect size and level of autocorrelation. No Phase A trends were present in the data. To reduce a very long running time the minimum number of baseline observations was set to 5 and each simulation condition was only repeated 2x 1000 times.

**Supplementary Table 3.**

***Estimated power of the three tests with Phase A trend present and 120 observations***


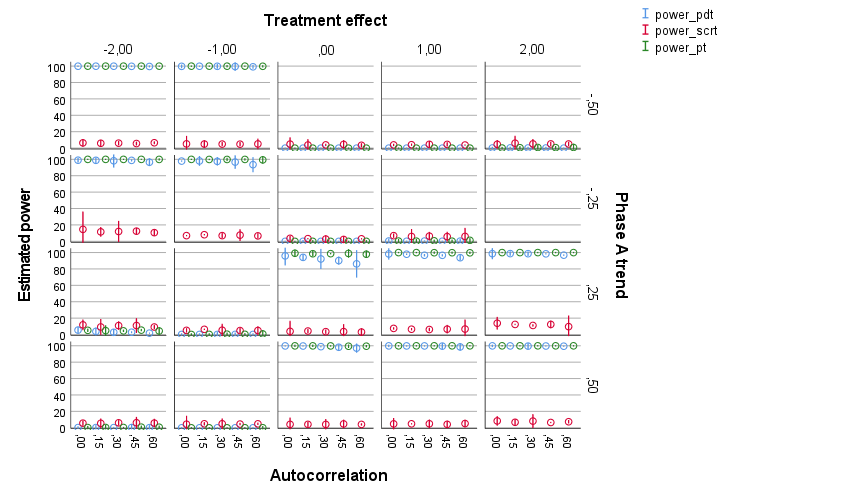


*Note.* Average estimated power (with 95% confidence interval) of the Permutation Distancing Test (blue), Single-Case Randomization Test (red), and traditional permutation test (green), with linear Phase A trends present in the data with 120 observations, per treatment effect size and level of autocorrelation. No Phase B trends were present in the data. To reduce a very long running time the minimum number of baseline observations was set to 5 and each simulation condition was only repeated 2x 1000 times.

**Supplementary Table 4.**

***Estimated power of the three tests with Phase B trend present and 120 observations***


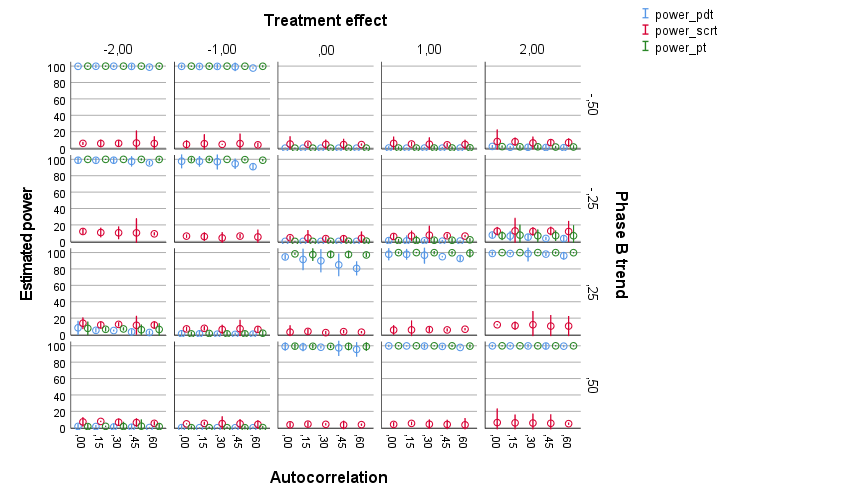


*Note.* Average estimated power (with 95% confidence interval) of the Permutation Distancing Test (blue), Single-Case Randomization Test (red), and traditional permutation test (green), with linear Phase B trends present in the data with 120 observations, per treatment effect size and level of autocorrelation. No Phase A trends were present in the data. To reduce a very long running time the minimum number of baseline observations was set to 5 and each simulation condition was only repeated 2x 1000 times.
